# Effectiveness of prehospital Magill forceps use for out-of-hospital cardiac arrest due to foreign body airway obstruction in Osaka City

**DOI:** 10.1186/s13049-014-0053-3

**Published:** 2014-09-04

**Authors:** Tomohiko Sakai, Tetsuhisa Kitamura, Taku Iwami, Chika Nishiyama, Kayo Tanigawa-Sugihara, Sumito Hayashida, Tatsuya Nishiuchi, Kentaro Kajino, Taro Irisawa, Tadahiko Shiozaki, Hiroshi Ogura, Osamu Tasaki, Yasuyuki Kuwagata, Atsushi Hiraide, Takeshi Shimazu

**Affiliations:** Department of Traumatology and Acute Critical Medicine, Osaka University Graduate School of Medicine, 2-15 Yamadaoka Suita, Osaka, 565-0871 Japan; Division of Environmental Medicine and Population Sciences, Department of Social and Environmental Medicine, Graduate School of Medicine, Osaka University, 2-2 Yamadaoka Suita, Osaka, 565-0871 Japan; Kyoto University Health Services, Yoshida-Honmachi, Sakyo-ku, Kyoto, 606-8501 Japan; Department of Critical Care Nursing, Kyoto University Graduate School of Human Health Science, 53 Shogoin Kawahara-cho, Sakyo-ku, Kyoto, 606-8507 Japan; Osaka Municipal Fire Department, 1-12-54 Kujo Minami, Nishi-ku, Osaka, 550-8566 Japan; Department of Acute Medicine, Kinki University Faculty of Medicine, 377-2 Ohno-Higashi Osaka-Sayama, Osaka, 589-8511 Japan; Department of Acute Madicine & Critical Care Medical Center, Osaka National Hospital, 2-1-14 Hoenzaka, Chuo-ku, Osaka, 540-0006 Japan; Nagasaki University Hospital Emegency Medical Center, 1-7-1, Sakamoto, Nagasaki, 852-8501 Japan; Department of Emergency and Critical Care Medicine, Kansai Medical University Hirakata Hospital, 2-3-1 Shinmachi, Hirakata, Osaka, 573-1191 Japan

**Keywords:** Foreign body airway obstruction, Magill forceps, Cardiac arrest, Emergency medical service

## Abstract

**Background:**

Although foreign body airway obstruction (FBAO) accounts for many preventable unintentional accidents, little is known about the epidemiology of FBAO patients and the effect of forceps use on those patients. This study aimed to assess characteristics of FBAO patients transported to hospitals by emergency medical service (EMS) personnel, and to verify the relationship between prehospital Magill forceps use and outcomes among out-of-hospital cardiac arrests (OHCA) patients with FBAO.

**Methods:**

We retrospectively reviewed ambulance records of all patients who suffered FBAO, and were treated by EMS in Osaka City from 2000 through 2007, and assessed the characteristics of those patients. We also performed a multivariate logistic-regression analysis to assess factors associated with neurologically favorable survival among bystander-witnessed OHCA patients with FBAO in larynx or pharynx.

**Results:**

A total of 2,354 patients suffered from FBAO during the study period. There was a bimodal distribution by age among infants and old adults. Among them, 466 (19.8%) had an OHCA when EMS arrived at the scene, and 344 were witnessed by bystanders. In the multivariate analysis, Magill forceps use for OHCA with FBAO in larynx or pharynx was an independent predictor of neurologically favorable survival (16.4% [24/146] in the Magill forceps use group versus 4.3% [4/94] in the non-use group; adjusted odds ratio, 3.96 [95% confidence interval, 1.21–13.00], *p* = 0.023).

**Conclusions:**

From this large registry in Osaka, we revealed that prehospital Magill forceps use was associated with the improved outcome of bystander-witnessed OHCA patients with FBAO.

## Background

Foreign body airway obstruction (FBAO) represents a true emergency [1]. In Japan, over 9,000 die of suffocation every year, and the incidence rate is approximately 7.4 per 100,000 person-years, the second most common form of death following traffic accidents in the group of unintentional injury mortality [2]. Although FBAO accounts for many preventable unintentional accidents [3,4], little is known about the incidence, characteristics, and outcomes of FBAO patients in prehospital emergency settings [1,3]. Chest thrusts, back blows, and abdominal thrusts are performed for emergency patients to relieve FBAO [4]. However, the effectiveness of Magill forceps, used by emergency medical service (EMS) personnel for FBAO patients, still has not been established. Its use might especially contribute to improving outcomes after out-of-hospital cardiac arrest (OHCA) with FBAO.

The Osaka Municipal Fire Department has registered the ambulance records in Osaka City, a large metropolitan community with approximately 2.6 million residents, and linked them to the data on resuscitation, simultaneously collected according to the Utstein style guidelines since January 2000 [5]. This study aims to assess incidence, characteristics and outcomes of FBAO patients transported to hospitals by EMS, and to verify the relationship between prehospital Magill forceps use and outcomes among OHCA patients with FBAO in a large urban community.

## Methods

### Target area study patients

The target area for this study was Osaka City in Japan, which has an urban area of 221 km^2^ and had a residential population of 2,598,774 as of 2000 (population density; approximately 11,700 persons/km^2^) [6]. Males make up 49.0% of the population, 17.1% of whom are 65 years old or older.

We retrospectively reviewed ambulance records of all patients who had FBAO in airways, including oral cavity, pharynx, larynx, trachea and bronchus, who were treated by EMS and then transported to medical institutions in Osaka City from January 1, 2000 through December 31, 2007. Diagnoses from the ambulance records were clinically determined by a physician in charge, working in collaboration with the EMS personnel. Approval for the study was obtained from the Ethics Committee of Kyoto University Graduate School of Medicine.

### EMS organization and equipment in Osaka

The 119 emergency telephone number is accessible anywhere in Japan, including Osaka City. Upon receiving a 119 call, an emergency dispatch center sends the nearest available ambulance to the site. In the year 2000, the Osaka Municipal Fire Department had a total of 50 EMS teams, one dispatch center, 25 fire stations, and 22 branches of fire stations [7]. Each ambulance consists of a 3-person unit providing life support 24 hours a day. To relieve FBAO, the trained EMS personnel are allowed to use Magill forceps in addition to finger sweep, back blows or slaps, abdominal thrusts, and chest thrusts. The size of Magill forceps used by EMS personnel in this study area was the same irrespective of patient age. Most highly-trained EMS personnel are called Emergency Life-Saving Technicians (ELSTs), the majority of whom are only allowed to insert an intravenous line and an adjunct airway, and to use a semi-automated external defibrillator for OHCA patients. Specially-trained ELSTs has been allowed to perform tracheal intubation since July 2004, and to administer epinephrine to only OHCA patients since April 2006 [8].

In order for EMS personnel in Japan to use Magill forceps in prehospital settings, fire department personnel must be an Emergency Medical Technician (EMT) or an ELST. To become an EMT, they are required to have received fundamental medical education in emergency care for 250 hours through a training academy. There are two options to becoming certified as an ELST in Japan [9]. The first is through the educational system within the fire department itself. After being actively engaged in pre-hospital setting as an EMT for more than 5 years or 2,000 hours, EMTs must pass the national examination of ELST after having basically received at least one additional year of medical education and training at the fire academy. The second way is through the education system in an accredited EMT school or college. To become an ELST, candidates must pass the national examination of ELST after receiving medical education and training in emergency care at the certified EMT school or college for at least two years.

### Data collection and quality control

The EMS ambulance record included patients’ age, gender, location of accident, region of foreign body, patients’ condition at time of EMS arrival, Magill forceps use, epinephrine, intubation, and ambulance time courses. If the patient had cardiopulmonary arrests when EMS arrived at the scene or during EMS treatments, data on the resuscitation course were collected using a data form according to the Utstein-style reporting guidelines [10,11]. Information on the type of bystander resuscitation attempts and activities of daily living (ADL) before arrest was obtained from bystanders by an EMS interview before leaving the scene. In this study, ADL before arrest was divided in the following categories; good (defined as having ability to perform common life without assistance by other persons), disability (other than good), and unclassified. EMS times of calls received, time of vehicle arrival at the scene, contact with patients, initiation of cardiopulmonary resuscitation (CPR), defibrillation by EMS, and hospital arrival time were recorded with a clock used by each EMS system. All survivors who suffered from OHCA were followed up for up to one month after the event by EMS personnel in charge. One month neurological outcomes were determined by the physician in charge, using the cerebral performance category (CPC) scale: category 1, good cerebral performance; category 2, moderate cerebral disability; category 3, severe cerebral disability; category 4, coma or vegetative state; and category 5, death [10,11]. Neurologically favorable survival was defined as a CPC category 1 or 2, no change from baseline CPC [10,11].

### Statistical analysis

The annual incidence per 100,000 inhabitants was calculated based on the population in the year 2000. The age-adjusted annual incidence of OHCAs per 100,000 inhabitants was calculated by direct methods using year 2000 census data and the Japanese model population of 1985 [2]. Patient characteristics were evaluated with *t*-test for numerical variables and a chi-square test for categorical variables. Trend tests for continuous variables were performed with the Spearman rank statistic method. To investigate between Magill forceps use and neurological outcomes after OHCA, we focused on bystander-witnessed OHCA patients with FBAO in larynx and pharynx, because it was possible to remove intraoral foreign bodies with finger sweep, but it would be inappropriate to remove trachea and bronchus foreign bodies with Magill forceps. Multivariable analysis was used to assess the contribution of Magill forceps use to neurologically favorable survival; odds ratios (ORs) and their 95% confidence intervals (CIs) were calculated. Potential confounding factors included gender, age, location of accident, ADL before arrest, bystander-initiated CPR, time interval from collapse to call and time interval from call to arrival at the hospital. All the tests were 2-tailed, and *p* values of <0.05 were considered statistically significant. All statistical analyses were performed using the SPSS statistical package version 16.0 J (IBM Corp. Armonk, NY).

This manuscript was written based on the STROBE statement to assess the reporting of cohort and cross sectional studies [12].

## Results

A total of 1,531,017 ambulance records were documented during the eight-year study period. Of these records, 2,354 (0.2%) were FBAO patients. The age-adjusted annual incidence of FBAO patients per 100,000 inhabitants are shown in Table 1. The age-adjusted incidence rates of all FBAO patients were 9.5 in 2000 and 9.5 in 2007, the incidence rates of OHCA when EMS arrived were 1.1 and 1.4, and rates of witnessed CPA were 0.9 and 1.1, respectively. The rates remained stable during the study period.

**Table 1:** **Temporal trends in age-adjusted incidence rates of FBAO patients**

**Year**	**2000**	**2001**	**2002**	**2003**	**2004**	**2005**	**2006**	**2007**	***p*** **for trend**
All FBAO patients									
Annual patients	252	299	293	309	319	296	301	285	
Incidence	9.7	11.5	11.3	11.9	12.3	11.4	11.6	11.0	
Age-adjusted incidence	9.5	10.1	9.8	10.6	10.9	9.8	9.6	9.5	0.690
OHCA when EMS arrived									
Annual patients	46	53	68	48	61	58	73	59	
Incidence	1.8	2.0	2.6	1.8	2.3	2.2	2.8	2.3	
Age-adjusted incidence	1.1	1.3	1.5	1.1	1.4	1.5	1.8	1.4	0.120
Witnessed OHCA when EMS arrived									
Annual patients	38	36	54	35	40	41	52	48	
Incidence	1.5	1.4	2.1	1.3	1.5	1.6	2.0	1.8	
Age-adjusted incidence	0.9	0.9	1.2	0.8	0.9	1.1	1.3	1.1	0.217

Data indicate age-adjusted annual incidence rates per 100,000 population per year.

*Abbreviations:* *FBAO* foreign body airway obstruction, *OHCA* out-of-hospital cardiac arrest, *EMS* emergency medical service.

**Figure 1:**
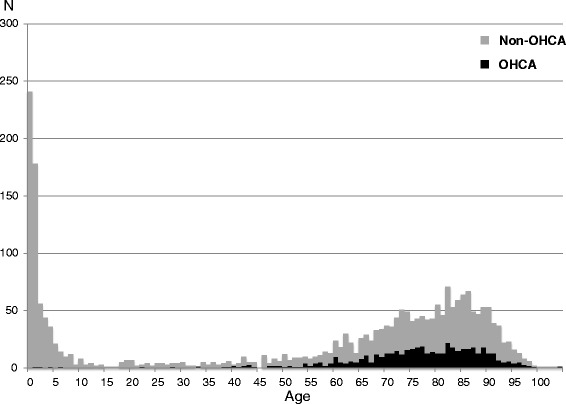
**Age distribution of FBAO patients transported to hospitals by EMS.** The black bars show OHCA patients, and gray bars show non-OHCA patients. FBAO: foreign body airway obstruction; EMS: emergency medical service; OHCA: out-of-hospital cardiac arrest.

The characteristics of FBAO patients are noted in Table 2. The mean age of all FBAO patients was 54.7 years and males were 50.8%. The age showed a bimodal distribution and its frequencies were high among infants and older adults (Figure 1). In particular, 241 (10.2%) were infants aged <1 year. Locations of accidents were at home (68.8%), in a health care facility (18.1%) and others (13.1%). Regions of foreign body were 15.0% in oral cavity, 64.8% in larynx or pharynx, and 20.2% in trachea or bronchus, respectively. Half of the FBAO patients did not have functional disorders in their respiration when EMS arrived at the scene, whereas 17.2% had dyspnea, 9.4% had breathing difficulties, 1.4% had respiratory arrest, and 19.8% had cardiopulmonary arrest. Prehospital Magill forceps was used to remove a foreign body from 383 patients (16.3%) at the scene.

**Table 2:** **FBAO patient characteristics throughout study period**

	**FBAO patients**
	**N = 2,354**
Age, year, mean ± SD	54.7 ± 35.0
Median (IQR)	71 (6–83)
Male, N (%)	1,195 (50.8)
Location of accident, N (%)	
Home	1,620 (68.8)
Health care facility	426 (18.1)
Others	308 (13.1)
Region of foreign body, N (%)	
Oral cavity	352 (15.0)
Pharynx or larynx	1,526 (64.8)
Trachea or bronchus	476 (20.2)
Patients’ condition when EMS arrival, N (%)	
Normal	1,230 (52.3)
Feel dyspnea	404 (17.2)
Breathing difficulty	221 (9.4)
Respiratory arrest	33 (1.4)
Cardiopulmonary arrest	466 (19.8)
Use of prehospital Magill forceps, N (%)	383 (16.3)

*Abbreviations:* *SD* standard deviation, *IQR* interquartile range, *EMS* emergency medical service.

**Figure 2:**
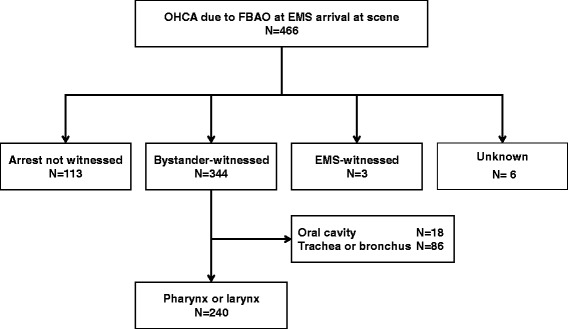
**Overview of EMS-treated FBAO patients with an abridged Utstein template (January 1, 2000 to December 31, 2007).** FBAO: foreign body airway obstruction; EMS: emergency medical service; OHCA: out-of-hospital cardiac arrest.

A total of 466 patients had OHCA with FBAO before hospital arrival, and 344 were witnessed by bystanders (Figure 2). Of them, 18 (5.2%) had foreign body in their mouth, 86 (25.0%) in trachea or bronchus and, 240 (69.8%) in larynx or pharynx. Characteristics and outcomes of bystander-witnessed OHCA with FBAO caused by pharyngeal or laryngeal obstruction with or without prehospital Magill forceps use are noted in Table 3. The forceps use group was more likely to be younger and to be at home, and was less likely to receive bystander-initiated CPR than the non-forceps group. Only one patient was child aged <18 years old. There were no significant differences in the male/female ratio, ADL before arrests, and ventricular fibrillation as first documented rhythm. Although the mean time interval from collapse to call was not different between the groups, the time interval from call to hospital arrival was significantly shorter in the non-forceps group than in the forceps use group. Neurologically favorable one-month survival among the forceps use group (16.4% [24/146]) was significantly higher than among the non-forceps group (4.3% [4/94], *p* = 0.004).

**Table 3:** **Characteristics and outcomes of bystander-witnessed OHCA patients caused by pharyngeal or laryngeal obstruction**

	**Forceps use**	**Non-use**	***p*** **value**
	**N = 146**	**N = 94**	
Age, year, mean ± SD	73.6 ± 12.9	78.1 ± 14.3	0.013
Male, N (%)	91 (62.3)	52 (55.3)	0.285
Location of accident, n (%)			
Home	107 (73.3)	54 (57.4)	
Health care facility	16 (11.0)	35 (37.2)	<0.001
Others	23 (15.8)	5 (5.3)	
Activities of daily living before arrests, N (%)			
Good	80 (54.8)	40 (42.6)	0.062
Disability	61 (41.8)	53 (56.4)	
Unclassified	5 (3.4)	1 (1.1)	
Ventricular fibrillation as first documented rhythm, N (%)	0 (0.0)	3 (2.1)	0.281
Bystander-initiated CPR, N (%)			
Compression-only CPR	8 (5.5)	16 (17.0)	
Conventional CPR	20 (13.7)	24 (25.5)	0.001
No CPR	117 (80.1)	54 (57.4)	
Epinephrine, N (%)	2 (3.6)	3 (6.8)	0.653
Intubation, N (%)	7 (4.8)	8 (8.5)	0.281
Time interval, min, mean ± SD			
Collapse to call	3.2 ± 5.5	3.1 ± 4.0	0.818
Call to EMS arrival at the scene	7.4 ± 2.4	7.2 ± 2.4	0.513
Call to arrival at the hospital	28.0 ± 7.6	24.7 ± 7.1	0.001
Outcomes			
Survival at one month, N (%)	39 (26.7)	16 (17.0)	0.086
Neurologically favorable one-month survival, N (%)	24 (16.4)	4 (4.3)	0.004

*Abbreviations:* *SD* standard deviation, *CPR* cardiopulmonary resuscitation, *EMS* emergency medical system.

In a multivariable analysis (Table 4), prehospital Magill forceps use for OHCA patients with FBAO in larynx or pharynx was an independent predictor of neurologically favorable one-month survival (adjusted OR, 3.96 [95% CI, 1.21–13.00], *p* = 0.023), and the time interval from collapse to call was also an independent predictor (adjusted OR, 0.87 [95% CI, 0.77–0.99], *p* = 0.032). Other factors did not contribute to better neurological outcome after adult bystander-witnessed OHCAs with FBAO.

**Table 4:** **Adjusted odds ratio of patient and EMS characteristics for neurologically favorable survival**

	**Adjusted OR**	**95% CI**		***p*** **value**
Age (for 1-increment of year)	0.98	0.95	1.02	0.308
Male	1.08	0.45	2.61	0.858
Location of accident				
Home	Reference			
Health care facility	1.73	0.40	7.52	0.465
Others	2.54	0.82	7.93	0.108
Disability in activities of daily living before arrests	1.04	0.41	2.65	0.930
Bystander-initiated CPR	0.73	0.22	2.41	0.605
Magill forceps use	3.96	1.21	13.00	0.023
Collapse to call (for 1-increment of minute)	0.87	0.77	0.99	0.032
Call to arrival at the hospital (for 1-increment of minute)	0.99	0.93	1.05	0.774

*Abbreviations:* *CPR* cardiopulmonary resuscitation, *EMS* emergency medical system, *OR* odds ratio, *CI* confidence interval.

## Discussion

From the extensive ambulance records including Utstein registry in a large urban city, this study showed the epidemiology of FBAO patients who were transported to hospitals by EMS, approximately 20% of whom resulted in OHCA. In addition, we revealed that prehospital Magill forceps use for bystander-witnessed OHCA patients with FBAO in larynx or pharynx was associated with the improved neurological outcome. To our knowledge, this is the first observation to demonstrate the effectiveness of Magill forceps for OHCA due to FBAO in prehospital settings. Our population-based registry covering 2.6 million people enabled us to assess the effects of prehospital Magill forceps, and may well provide helpful information to improve prehospital care worldwide.

This study underscored that prehospital Magill forceps use was associated with better outcome after OHCA due to FBAO. Although Magill forceps is a tool for removing foreign bodies [1], its effectiveness has not been sufficiently investigated. In the prehospital settings in Japan, only trained EMS personnel were allowed to use Magill forceps to relieve airway obstruction [13]. In this study, approximately two-thirds of OHCA patients with FBAO had foreign bodies in larynx or pharynx. Therefore, EMS personnel should proactively observe OHCA patients’ larynx or pharynx in case of suspected FBAO, by interviews with bystanders or their surroundings when they arrive at the scene, and try to remove foreign bodies by using Magill forceps. By showing the effectiveness of prehospital Magill forceps for OHCA patients with FBAO, this study suggests that EMS personnel should receive training in the use of Magill forceps in prehospital settings. In the EMS system of Japan, Magill forceps by EMS personnel has been recommended and used to relieve FBAO in prehospital settings. On the other hand, to further improve the outcomes after OHCA due to FBAO, new device use such as video laryngoscope for detection and release of FBAO might be of help [14], and further efforts to save lives of these patients are essential.

In Osaka City, the age-adjusted annual incidence per 100,000 inhabitants of FBAO patients transported to hospitals by EMS was approximately 10.0, and the incidence of OHCA with FBAO when EMS arrived at the scene was approximately 1.5. In preceding studies, the incidence of unintentional suffocation per 100,000 inhabitants was more than 6 in France [15] and 3.3% of FBAO patients died of it in San Diego [1]. The incidence and mortality among FBAO patients therefore seemed to differ by region. These differences might be explained partially by dietary habits and life-style [16]. In any case, these differences require further study.

The age distribution of emergency FBAO patients in this study had bimodality among infants and older adults, a result consistent with previous studies [1,3,17]. The reason for this bimodality would be that older adults have difficulty in swallowing and coughing up as they increasing age [18], and infants tend to put objects such as toys, coins, pen caps, and clips in their mouths [19]. Although there might be differences in causes of FBAO between infants and older adults, FBAO is, most importantly, a preventable accident [16]. Therefore, to prevent death from FBAO, mothers should pay attention to their babies’ behaviors and family members and healthcare facility staff must provide meals appropriate for older adult swallowing function. Furthermore, it would be important to engage in educational activities on the prevention of FBAO for the general public, because the annual incidence of FBAO in our study area did not decrease during the study period.

When bystanders encounter FBAO patients, their first aid response is very important. As methods to remove for removing foreign bodies from FBAO patients, 5 back blows following 5 chest thrusts for infants with FBAO, and chest thrusts, back slaps, and abdominal thrusts for unresponsive adults with FBAO are recommended in the CPR guidelines [4,20]. In addition, an earlier call was an independent predictor of better outcome after OHCA due to FBAO in a multivariate analysis. Therefore, this result would reinforce the importance of an early call in the chain of survival, and suggests that activating the EMS system quickly leads to improving outcomes from OHCA due to FBAO. However, because evidence concerning the epidemiology and outcomes from FBAO are scarce, further efforts to collect population-based data on FBAO (as in this study) in various countries would lead to improving outcomes.

### Limitation

This study has some inherent limitations. First, data are lacking regarding what type of foreign bodies caused of airway obstruction, and whether they were removed or not in the prehospital settings. Second, this study enrolled only emergency patients transported by EMS and did not obtain information on those who went to the hospital directly by themselves. Another limitation was that this study was a retrospective observational study, and an association between prehospital Magill forceps and the outcomes after OHCA due to FBAO should, therefore, be confirmed by other cohorts or randomized controlled trials. Finally, there might be unmeasured confounding factors influencing the association between prehospital Magill forceps use and outcomes after OHCA due to FBAO.

## Conclusion

In the large metropolitan community of Osaka in Japan, our study demonstrated the epidemiology of FBAO patients transported to hospitals by EMS, finding that approximately 20% of FBAO patients resulted in OHCA in this area. In addition, we revealed that prehospital Magill forceps use was associated with the improved neurological outcome for bystander-witnessed OHCA patients with FBAO in larynx or pharynx. Further investigations by other cohorts or randomized controlled trials are needed to confirm these associations.

## Abbreviations

FBAO: Foreign body airway obstruction
OHCA: Out-of-hospital cardiac arrest
EMS: Emergency medical service
CPR: Cardiopulmonary resuscitation
ELST: Emergency Life-Saving Technician
ADL: Activities of daily living
CPC: Cerebral performance category
OR: Odds ratio
CI: Confidence interval

## References

[1] Soroudi A, Shipp HE, Stepanski BM, Ray LU, Murrin PA, Chan TC, Davis DP, Vilke GM (2007). Adult foreign body airway obstruction in the prehospital setting. Prehosp Emerg Care.

[2] Japan National Statistics Center: **Population Dynamics of Japan.** 2000, [http://www.e-stat.go.jp/SG1/estat/GL08020103.do?_csvDownload_&fileId=000006552899&releaseCount=3] (Accessed May 19, 2014) (in Japanese).

[3] Vilke GM, Smith AM, Ray LU, Steen PJ, Murrin PA, Chan TC (2004). Airway obstruction in children aged less than 5 years: the prehospital experience. Prehosp Emerg Care.

[4] Sayre MR, Koster RW, Botha M, Cave DM, Cudnik MT, Handley AJ, Hatanaka T, Hazinski MF, Jacobs I, Monsieurs K, Morley PT, Nolan JP, Travers AH, Adult Basic Life Support Chapter Collaborators (2010). Part 5: adult basic life support: 2010 International Consensus on Cardiopulmonary Resuscitation and Emergency Cardiovascular Care Science With Treatment Recommendations. Circulation.

[5] Irisawa T, Iwami T, Kitamura T, Nishiyama C, Sakai T, Tanigawa-Sugihara K, Hayashida S, Nishiuchi T, Shiozaki T, Tasaki O, Kawamura T, Hiraide A, Shimazu T: **An association between systolic blood pressure and stroke among patients with impaired consciousness in out-of-hospital emergency settings.** *BMC Emerg Med* 2013, **13:**24. 10.1186/1471-227X-13-24 PMC3878578 24341562

[6] Statistic Bureau, Ministry of Internal Affairs and Communications: **Population Census of Japan.** 2000, [http://www.city.osaka.lg.jp/toshikeikaku/cmsfiles/contents/0000015/15577/H12-kokuseityousa1ji-1.xls] (Accessed May 19, 2014) (in Japanese).

[7] Osaka Municipal Fire Department (2007). Osaka Municipal Emergency Annual Report.

[8] Tanigawa K, Tanaka K (2006). Emergency medical service systems in Japan: past, present, and future. Resuscitation.

[9] Kajino K, Kitamura T, Iwami T, Daya M, Ong ME, Nishiyama C, Sakai T, Tanigawa-Sugihara K, Hayashida S, Nishiuchi T, Hayashi Y, Hiraide A, Shimazu T (2014). Impact of the number of on-scene emergency life-saving technicians and outcomes from out-of-hospital cardiac arrest in Osaka City. Resuscitation.

[10] Cummins RO, Chamberlain DA, Abramson NS, Allen M, Baskett PJ, Becker L, Bossaert L, Delooz HH, Dick WF, Eisenberg MS, Evans TR, Holmberg S, Kerber R, Mullie A, Ornato JP, Sandoe E, Skulberg A, Tunstall-Padoe H, Swanson R, Thies WH (1991). Recommended guidelines for uniform reporting of data from out-of-hospital cardiac arrest: the Utstein Style. A statement for health professionals from a task force of the American Heart Association, the European Resuscitation Council, the Heart and Stroke Foundation of Canada, and the Australian Resuscitation Council. Circulation.

[11] Jacobs I, Nadkarni V, Bahr J, Berg RA, Billi JE, Bossaert L, Cassan P, Coovadia A, D’Este K, Finn J, Halperin H, Handley A, Herlitz J, Hickey R, Idris A, Kloeck W, Larkin GL, Mancini ME, Mason P, Mears G, Monsieurs K, Montgomery W, Morley P, Nichol G, Nolan J, Okada K, Perlman J, Shuster M, Steen PA, Sterz F (2004). Cardiac arrest and cardiopulmonary resuscitation outcome reports: update and simplification of the Utstein templates for resuscitation registries: a statement for healthcare professionals from a task force of the International Liaison Committee on Resuscitation (American Heart Association, European Resuscitation Council, Australian Resuscitation Council, New Zealand Resuscitation Council, Heart and Stroke Foundation of Canada, InterAmerican Heart Foundation, Resuscitation Councils of Southern Africa). Circulation.

[12] Von Elm E, Altman DG, Egger M, Pocock SJ, Gotzsche PC, Vandenbroucke JP, STROBE Initiative (2008). The Strengthening the Reporting of Observational Studies in Epidemiology (STROBE) statement: guidelines for reporting observational studies. J Clin Epidemiol.

[13] Fire and Disaster Management Agency of Japan: **Criteria on Prehospital Emergency Treatments by EMS Personnel.** [http://www.fdma.go.jp/concern/law/kokuji/hen52/52050000020.htm] (Accessed May 20, 2014) (in Japanese).

[14] Mifflin KA, Richenberger E, Horning J, Gleisberg GR, Escot ME, Monroe BJ (2013). Seeing the difference: using video laryngoscopes for foreign body airway obstruction. JEMS.

[15] Fingerhut LA, Cox CS, Warner M (1998). International comparative analysis of injury mortality. Findings from the ICE on injury statistics. International Collaborative Effort on Injury Statistics. Adv Data.

[16] Higuchi O, Adachi Y, Ichimaru T, Asai M, Kawasaki K (2009). Foreign body aspiration in children: a nationwide survey in Japan. Int J Pediatr Otorhinolaryngol.

[17] Inamasu J, Miyatake S, Tomioka H, Shirai T, Ishiyama M, Komagamine J, Maeda N, Ito T, Kase K, Kobayashi K (2010). Cardiac arrest due to food asphyxiation in adults: resuscitation profiles and outcomes. Resuscitation.

[18] Wick R, Gilbert JD, Byard RW (2006). Cafe coronary syndrome-fatal choking on food: an autopsy approach. J Clin Forensic Med.

[19] Andazola JJ, Sapien RE (1999). The choking child: what happens before the ambulance arrives?. Prehosp Emerg Care.

[20] Berg MD, Schexnayder SM, Chameides L, Terry M, Donoghue A, Hickey RW, Berg RA, Sutton RM, Hazinski MF (2010). Part 13: pediatric basic life support: 2010 American Heart Association Guidelines for Cardiopulmonary Resuscitation and Emergency Cardiovascular Care. Circulation.

